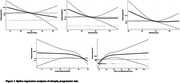# Markers of atrophy progression in patients with young‐onset cognitive complaints not due to Alzheimer’s Disease

**DOI:** 10.1002/alz.091197

**Published:** 2025-01-09

**Authors:** Chiara Carbone, Erica Balboni, Ludovico Luchetti, Teresa Urbano, Daniela Beltrami, Riccardo Maramotti, Manuela Tondelli, Chiara Gallingani, Federico Gasparini, Giulia Vinceti, Alessandro Marti, Annalisa Chiari, Giovanna Zamboni

**Affiliations:** ^1^ Università di Modena e Reggio Emilia, Modena Italy; ^2^ Fisica Medica, Azienda Ospedaliero Universitaria di Modena, Modena Italy; ^3^ Università di Siena, Siena Italy; ^4^ CREAGEN ‐ Centro di Ricerca in Epidemiologia Ambientale, Genetica e Nutrizionale, Università di Modena e Reggio Emilia, Modena Italy; ^5^ Neuropsicologia Clinica, Disturbi cognitivi e Dislessia nell'adulto, Azienda Unità Sanitaria Locale di Reggio Emilia‐IRCCS, Reggio Emilia Italy; ^6^ Neurologia, Azienda Ospedaliero Universitaria di Modena, Modena Italy

## Abstract

**Background:**

In clinical settings, the prognosis of patients with subtle cognitive complaints and no imaging evidence of neurodegeneration is often challenging, especially in conditions unrelated to Alzheimer’s Disease (AD). We aimed to identify which baseline indicators can help in the clinical decision‐making process of patients with Subjective Cognitive Decline (SCD) and Mild Cognitive Impairment (MCI) without AD pathology, by identifying those with faster brain atrophy for their age.

**Method:**

Young‐onset SCD and MCI patients (symptoms ≤65yo) were recruited. At baseline we administered neuropsychological assessment and anxiety/depression questionnaires to patients, while the Neuropsychiatric Inventory for behavioural symptoms to caregivers. Patients also underwent Magnetic Resonance Imaging (MRI) and serum neurofilaments light‐chain (NfLs) measurement, other than a (^18^F)Flutemetamol‐PET (amy‐PET) or lumbar puncture for collection of cerebrospinal fluid (CSF) NfLs, b‐amyloid_1‐42_, b‐amyloid_1‐42/1‐40_, total‐, and phosphorylated‐tau. After 18 months, MRI was repeated. Single‐subject percentage of brain volume change was calculated using the FMRIB Software Library tool SIENA and compared to the longitudinal change observed in healthy controls (Battaglini et al., 2019). Patients exceeding the 95^th^ percentile of normative values were categorized as having non‐physiological progressive atrophy. All variables and biomarkers were added in logistic and spline regression analyses with age, sex, education, and global cognitive status (i.e., Mini‐Mental State Examination – MMSE) as covariates, performed with Stata18.

**Result:**

Among the 75 patients recruited, 20 were excluded because of a CSF/amy‐PET suitable for AD; of the remaining 55, atrophy progressed in 18 while 37 had physiological brain volume changes. Low baseline performance in attentional‐executive (i.e., Frontal Assessment Battery) and language (i.e., phonemic, semantic, alternate fluency) tests were predictors of progressive atrophy, over and above baseline MMSE and demographical variables. Furthermore, caregiver‐reported apathy, along with elevated levels of serum NfLs, were indicative of a greater‐than‐expected change in brain volume after 18 months.

**Conclusion:**

In the assessment of young‐onset SCD and MCI patients, poor performance on attentional‐executive and language tests and high levels of serum NfLs, combined with apathy, should be considered markers for faster progression of brain atrophy in the short‐term future (18‐months), over and above the global cognitive status. Patients with these features should be monitored closely.